# Predictive role and clinical correlation of copeptin in patients with type 2 diabetes mellitus associated nephropathy approaching end-stage renal disease

**DOI:** 10.1186/s12882-026-04777-5

**Published:** 2026-01-30

**Authors:** Tooba Noor, Zareen Kiran, Muhammad Tassaduq Khan, Farina Hanif

**Affiliations:** 1 Department of Physiology, Dow University of Health Sciences, OJHA Campus, SUPARCO Road, Karachi, 75270 Pakistan; 2 National Institute of Diabetes and Endocrinology, Dow University of Health Sciences, OJHA Campus, SUPARCO Road, Karachi, 75270 Pakistan; 3 Department of Nephrology, Dow University Hospital, Dow University of Health Sciences, OJHA Campus, SUPARCO Road, Karachi, 75270 Pakistan; 4 Department of Biochemistry, Dow International Medical College, Dow University of Health Sciences, OJHA Campus, SUPARCO Road, Karachi, 75270 Pakistan

**Keywords:** Arginine vasopressin, Albuminuria, Copeptin, Diabetic nephropathy, End stage renal disease, Glomerular filtration rate and type 2 diabetes mellitus.

## Abstract

**Background:**

Diabetic Kidney Disease (DKD) is the leading cause of End Stage Renal Disease (ESRD). Arginine Vasopressin (AVP) plays pivotal roles in osmoregulation and renal water conservation. As patients with DKD are prone to develop osmotic irregularities, AVP is therefore, a potential pathophysiological target for disruption. Copeptin, a surrogate marker of AVP, is preferred over AVP due to comparatively greater stability and longer half-life. The study is designed to correlate the copeptin levels among subjects with Type 2 Diabetes Mellitus (DM) with worsening renal outcomes.

**Methods:**

It was a comparative cross-sectional study on 120 subjects with T2DM who were stratified into progressive deteriorating stages of chronic kidney disease (CKD) as per NKF KDOQI guidelines. Different biochemical variables HbA1c, serum BUN, serum creatinine UACR and GFR were done from the affiliated lab. Serum copeptin levels were determined using Copeptin sandwich ELISA technique. Data was analyzed through Statistical Program for Social Sciences (SPSS).

**Results:**

Out of the 120 subjects that were recruited for study, 30% of subjects (*n* = 36) developed severely decreased kidney function with GFR less than 30mL/min/1.73m2. Copeptin levels were seen to be increased with progressive stages of albuminuria (175.8 ± 148.4 pg/ml, 221.2 ± 213.1 pg/ml and 385.3 ± 288.4 pg/ml at UACR stage 1, 2 and 3, respectively) with positive correlation i.e. *r* = 0.375, p = ≤ 0.001. It was also found to be negatively correlated with declining GFR i.e. from 195.8 ± 174.1 pg/ml at stage 1 CKD to 291.7 ± 268.8 pg/ml at stage 5 CKD (*r* = -0.409, p = ≤ 0.001).

**Conclusions:**

Increase in serum copeptin levels from stage 1 to stage 5 of CKD suggest its predictive role in T2DM associated nephropathy, supporting its potential role as an early biomarker. Early detection may help delay ESRD progression through timely interventions.

**Supplementary Information:**

The online version contains supplementary material available at 10.1186/s12882-026-04777-5.

## Background

Diabetic Kidney Disease (DKD) is the most common micro vascular complications of Type 2 Diabetes Mellitus (T2DM) [1]. Approximately, 30–40% of individuals with T2DM develop DKD [2] leading to End Stage Renal Disease (ESRD). As per Kidney Disease: Improving Global Outcomes, 2022 Clinical Practice Guidelines for Diabetes Management in Chronic Kidney Disease (CKD), CKD is defined as persistent eGFR < 60 mL/min/1.73 m² along with Albumin Creatinine Ratio ≥ 30 mg/g that persist for at least 3 months [3]. The presentation may range from albuminuria to complete renal failure; progressing to ESRD requiring either dialysis or transplantation [4, 5]. Several important mechanisms are involved in the pathophysiology of T2DM and DKD; Arginine Vasopressin (AVP) system is one of them.

AVP is a nine amino acid containing hormone released from the posterior pituitary gland in response to increased plasma osmolarity or decreased plasma volume [6]. Physiologically, AVP causes arteriolar constriction mediated through V1a receptors and anti-diuresis via V2 receptors [7, 8].

AVP has pivotal role in osmoregulation, circulation and body fluid hemostasis. However, certain technical difficulties are associated with this hormone when it comes to the estimation of its levels in circulation. Its small size, short plasma half-life, unstable structure and binding with platelets in circulation make the reliable measurement of AVP difficult [9] and hinder its use as a biomarker commercially. In contrast to this, copeptin, a carboxyl terminal peptide of pre pro vasopressin, released in equimolar amount to that of AVP and equally respond rapidly to changes in osmolality as that of AVP, and also considered as more stable and sensitive surrogate biomarker of AVP [10–13].

Regarding renal dysfunction, several urinary and serum biomarkers are available but only a few of them are indicative of DKD. Creatinine is considered as a widely used indicator of chronic renal failure either due to kidney disease or multi organ failure and cannot differentiate between the origin of problem either hemodynamically mediated changes (pre-renal) or obstructive uropathy (post renal) [14]. Urinary Albumin Excretion (UAE) levels are limited to investigate the severity of proteinuria for diseases causing protein loss and to differentiate between tubule interstitial and glomerular diseases and so that for Urinary Albumin to Creatinine Ratio (UACR) [15]. But Copeptin has also been associated with metabolic syndrome, pre-diabetes, PCOS, and cardiovascular disease and their relevant complications [16]. Although copeptin and AVP concentrations are correlated well but their relationship distorted in CKD suggesting incomplete clearance of peptide, whenever the renal function is impaired [17]. Therefore, copeptin is preferred over AVP [18]. Also, it may not only act as predictor of early nephropathy but treatment strategy can also be targeted [12, 19] and [16].

Previous studies on copeptin have found its association with age, gender, incident type 2 diabetes mellitus, metabolic biomarkers of obesity and metabolic syndrome, cardiovascular morbidity and mortality associated with T2DM, albuminuria and declining GFR [12, 13, 19, 20–23]. We also previously showed that copeptin can be a useful marker in predicting transition in individuals from prediabetes to diabetes [24]. Despite these associations, it remains unknown whether copeptin can predict different stages of CKD in T2DM, particularly in South Asia, highlighting the novelty of the present study.

The outcome of this study will help to answer this question. Thus, the present study is designed to reveal trend of changes in copeptin levels from controls to different stages of CKD with deteriorating GFR and progressive albuminuria secondary to T2DM associated DKD to find a better and reliable biomarker to modulate treatment strategies that can delay the progression of ESRD.

## Methods

### Study design and subject recruitment

This comparative cross-sectional study recruited 120 participants via non stratified random sampling from the outpatient clinic of Diabetes and Nephrology from National Institute of Diabetes and Endocrinology, Dow University Hospital, Dow University of Health Sciences during July to December 2019. The sample size of at least 108 subjects was calculated using Open Epi.com Version 3 based on 90% power at 95% Confidence Interval and 5% margin of error, the mean ± standard deviation in diabetic and non-diabetic group was 55.5 ± 3.6 pg/dl and 3.22 ± 1.8 pg/dl, respectively from an estimated population of 45 [25]. However, it was rounded up to 120. Age- and gender-matched, normotensive, non-diabetic adults with no major comorbidities were part of controls whereas cases were T2DM patients who were controlled hypertensive (i.e. target B.*P* < 140/90mmHg and were on appropriate anti-hypertensive drugs). All parameters and comparative variables evaluated in the study were according to the guidelines as recommended by KDIGO 2022 CKD guidelines [2]. Ethical approval was obtained (IRB-1145/DUHS/Approval/2018), and all procedures conformed to the Declaration of Helsinki.

### Clinical data and biochemical reports

Individuals were stratified into progressive deteriorating stages of nephropathy based on the levels of UACR for advancing albuminuria and GFR for decline in renal function as per NKF KDOQI guidelines. After giving consent, the participants were inquired about their basic demographics such as age, gender, ethnicity, marital status, family history of diabetes mellitus, any other current medication for diabetes mellitus and hypertension.

Routine biochemical parameters like HbA1c were done by spectrophotometry on COBAS ROCHE c311 while, serum BUN, serum/ urinary creatinine, and urinary albumin excretion was measured on Abbot Architect c800 using kinetic, Jaffe’s and photometric method, respectively. Spot urinary albumin creatinine ratio (UACR) was calculated as spot urinary albumin divided by creatinine, expressed in mg/g. The results are expressed in mg/g to meet the standard guidelines. Estimated GFR was calculated using standardized Global Chronic Kidney Disease – Epidemiology Collaboration (CKD-EPI) equation. Copeptin levels in serum were determined in duplicates using Copeptin sandwich ELISA (Enzyme Linked Immunosorbent Assay) kit from Cloud Clone, China according to manufacturer protocol. The minimum detection limit of copeptin levels in this assay was 6.3 pg/mL. The functional sensitivity of intra assay coefficient variation was less than 10% while functional sensitivity of inter assay coefficient variation was less than 12% [24].

### Statistical analysis

Data was analyzed through Statistical Program for Social Sciences (SPSS) version 21. Normal distribution of variables was analyzed with Shapiro-Wilk test (*p* ≥ 0.05). Continuous variables were expressed as mean, standard deviation (SD) or median and ranges. One-way Analysis of Variance (ANOVA) was applied for comparison between various different groups and multiple comparison Dunnett test was applied for comparisons in between the groups. Pearson correlation was used to show the strength of relation between copeptin and affected factors followed by multivariate linear regression model and Receiver Operating Characteristics Curve (ROC) analysis. All statistical analysis was done at 95% confidence interval keeping the level of significance 5%.

## Results

### Baseline features of the study subjects

In total, 120 subjects were recruited for the study. Among them 53.3% were female. The average age of the selected sample population was 52.53 ± 11.4 years. The mean age at the time of diagnosis of T2DM was 47.27 ± 9.6 years whereas the average duration since the diagnosis of T2DM was 8.02 ± 7.4 years.

The mean weight of the study population was found to be 72.42 ± 12.8 kg, height (1.66 ± 0.1 m) and BMI (26.5 ± 4.7 kg/m^2^) at 95% confidence interval. Among the subjects, 30.8% (*n* = 37) were overweight (BMI 25–29.9 kg/m^2^) followed by 13.3% (*n* = 16) and 6.7% (*n* = 8) who were obese (BMI 3–34.9 kg/m^2^) and morbidy obese (BMI ≥ 35 kg/m^2^), respectively. The mean arterial pressure (MAP) was 107.7 ± 16.8 mmHg and 54.2% of the subjects had positive family history of DM. Hypertension (53.4%) was found to be the most common associated comorbidity followed by Benign Prostate Hyperplasia which was 8.3% prevalent in the study.

Approximately, half of the study population (49.2%) were taking anti-hypertensive drugs, out of them, ACE inhibitors/ARB were the most prescribed anti-hypertensive (13.3%) followed by Ca+-channel blockers (10.8%), the remaining 25% of subjects were on diuretics, centrally acting anti-hypertensive, beta blockers and combination drugs. Similarly, more than half of subjects were on anti-diabetic drugs (68.2%), with 27.5% were on oral hypoglycemic, 31.6% were on insulin and remaining 9.1% were on combination of insulin and oral hypoglycemic. Further details can be seen in our previously published paper [24, 26].

In this study, approximately 65% (*n* = 78/120) of the study population had nephropathy, out of them 21.67% (*n* = 26/120) had GFR < 30 mL/min/1.73 m² = G4–G5 along with Macroalbuminuria i.e. ≥ 300 mg/gm. The stratification of the study population as per the decline in renal functions are detailed in Table 1.

**Table 1:**
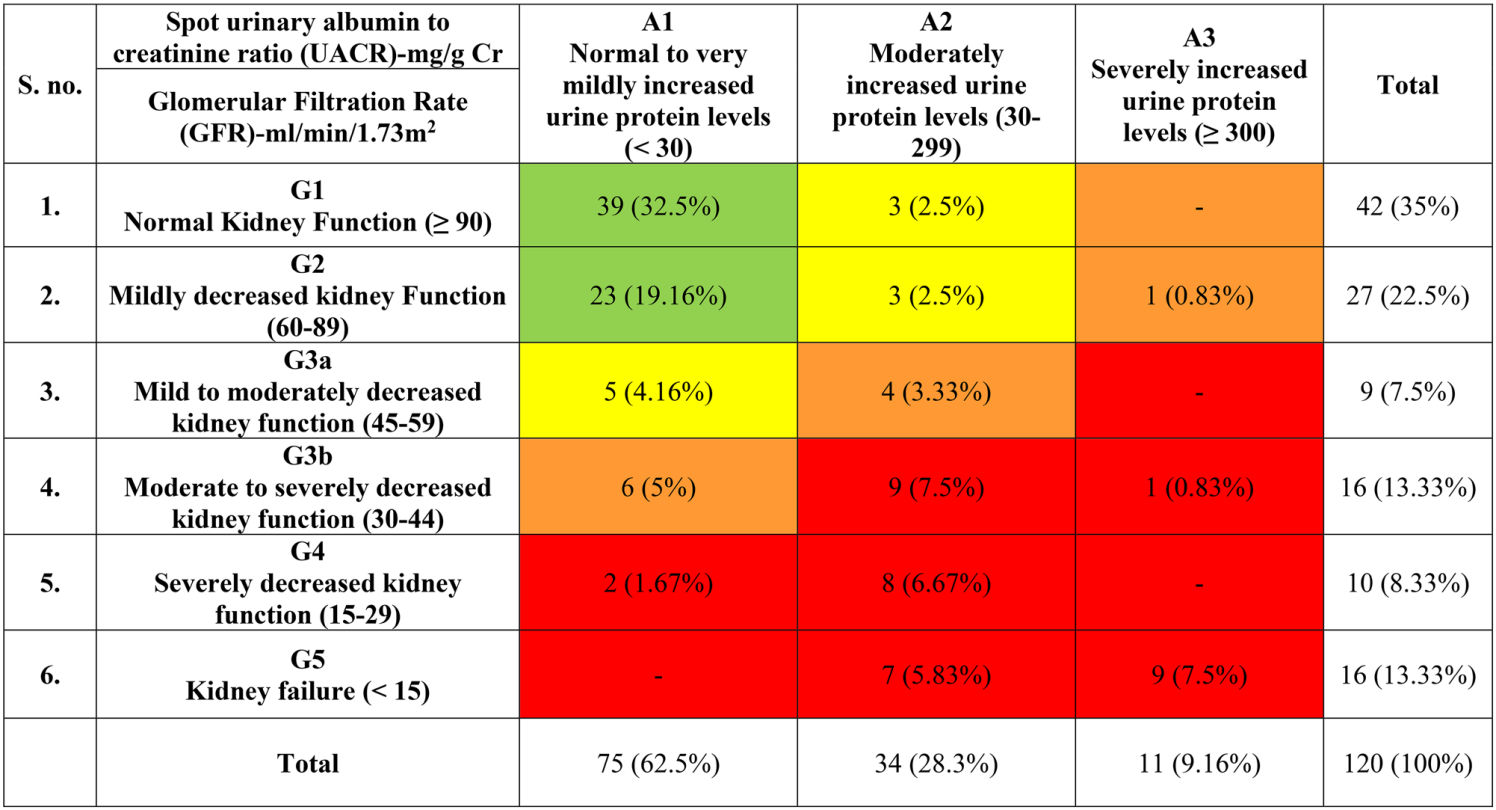
Stratification of study participants into different stages as per decline in renal function

### Stages of albuminuria in study population and its association with different biochemical variables

The recruited study population was stratified into different groups based on progressive stages of albuminuria depending on the levels of UACR. Stage 1 (Normoalbuminuria - UACR 3–30 mg/gm.), Stage 2 (Microalbuminuria – UACR 30–300 mg/gm.) and Stage 3 (Macroalbuminuria – UACR ≥ 300 mg/gm.). When the subjects were compared with different biochemical variables and copeptin, it was found out that copeptin levels raised more than two-fold between stage 1 (175.8 ± 148.4 pg/ml) to stage 3 of albuminuria (385.3 ± 288.4 pg/ml). Levels of copeptin were also found to be significantly different among different stages when ANOVA (F = 6.357, *p* = 0.002) and post-hoc Dunnett test was applied. The details of these variables in respective stages of albuminuria are given in Table 2.

**Table 2:** Changes in the levels of different biochemical variables and serum copeptin levels with progressive stages of albuminuria

S. no.	Variables	Spot Urinary Albumin Creatinine Ratio (mg/gm)			ANOVA	
		Stage 1 Normoalbuminuria-3-30 mg/gm	Stage 2 Microalbuminuria-30-300 mg/gm	Stage 3 Macroalbuminuria- >300 mg/gm	F- value	*p*-value
1.	HbA1c (%)	6.9 ± 1.7	8.6 ± 1.9***	9.3 ± 2.6***	13.7	≤ 0.001
2.	BUN (mg/dl)	29.3 ± 23.1	74.2 ± 47.2***	127.0 ± 63.9***	44.5	≤ 0.001
3.	Cr (mg/dl)	1.0 ± 0.5	2.7 ± 2.1***	5.5 ± 2.4***	57.9	≤ 0.001
4.	UAE (mg/l)	28.1 ± 36.9	590.2 ± 350.3***	3492.6 ± 1706.1***	203.0	≤ 0.001
5.	e GFR (ml/min/1.73m^2^)	85.8 ± 25.8	37.5 ± 27.4***	16.3 ± 22.4***	62.4	≤ 0.001
6.	Copeptin (pg/ml)	175.8 ± 148.4	221.2 ± 213.1	385.3 ± 288.3***	6.3	0.002

Where, ***Indicates significant difference between stage 1 and other stages of albuminuria i.e. *p* ≤ 0.001. All statistical tests were applied at 95% confidence interval. HbA1c = Glycosylated Hemoglobin, BUN = Blood Urea Nitrogen, Cr = Creatinine, UAE = Urine Albumin Excretion, e GFR = Estimated Glomerular Filtration Rate

### Stages of kidney failure in study population and its association with different biochemical variables

Study population was also segregated into following different stages of kidney disease; based on the decline of renal function estimated through eGFR. Diabetic and renal function biomarkers along with copeptin were also compared among these groups which are detailed in Table 3. The highest levels of HbA1c were recorded in stage 3b i.e. moderately to severely decreased kidney function which was 9.11 ± 1.7% with significant difference between stage 1 and stage 3b when ANOVA and post hoc multiple comparison Dunnett was applied (F = 3.651, *p* = 0.004). The mean serum copeptin levels of study population ranged from 195.8 ± 174.10 pg/ml in stage 1 to 291.7 ± 268.8 pg/ml to stage 5 while the individual levels of copeptin range from 18.2 pg/ml to 853.1 pg/ml. There was significant difference in copeptin levels when ANOVA was applied (F = 3.352, *p* = 0.007) at 95% confidence interval.

**Table 3:** Changes in the levels of different biochemical variables and serum copeptin levels with progressive decline in renal function

S. no.	Biochemical Variables		HbA1c (%)	BUN (mg/dl)	Creatinine (mg/dl)	UAE (mg/l)	UACR (mg/gm)	Copeptin (pg/ml)
	Stage of Kidney Disease GFR (ml/min/1.73m^2^)							
1.	Normal Kidney Function (≥ 90)		6.9 ± 1.8	23.2 ± 13.5	0.7 ± 0.1	57.3 ± 151.3	7.8 ± 20.2	195.8 ± 174.1
2.	Mildly decreased kidney Function (60–89)		7.3 ± 1.9	28.4 ± 9.5	0.9 ± 0.1	216.4 ± 698.5	42.1 ± 133.6	125.4 ± 84.2
3.	Mild to moderately decreased kidney function (45–59)		7.7 ± 2.1	31.9 ± 14.5	1.2 ± 0.1	251.6 ± 277.1	42.1 ± 47.5	365.3 ± 244.8
4.	Moderate to severely decreased kidney function (30–44)		9.1 ± 1.7 ***	60.3 ± 36.0 ***	1.8 ± 0.3 ***	541.0 ± 576.0	92.6 ± 99.1	176.2 ± 139.0
5.	Severely decreased kidney function (15–29)		8.5 ± 2.2	91.0 ± 38.9 ***	2.8 ± 0.6 ***	454.8 ± 351.9	105.1 ± 60.9	254.3 ± 246.5
6.	Kidney failure (< 15)		7.8 ± 2.1	137.9 ± 51.1 ***	6.4 ± 1.5 ***	2304.8 ± 2086.7***	1643.2 ± 1953.2***	291.7 ± 268.8
	ANOVA	F- value	3.6	51.1	214.5	16.8	14.1	3.3
		p-value	0.004	≤ 0.001	≤ 0.001	≤ 0.001	≤ 0.001	0.007

***Shows significant difference in the levels of biochemical variables in respective groups with controls i.e. *p* ≤ 0.001. All differences were evaluated at 95% confidence interval. Where, HbA1c = Glycosylated Hemoglobin, BUN = Blood Urea Nitrogen, UAE = Urine Albumin Excretion, UACR = Urine Albumin to Creatinine Ratio and GFR = Glomerular Filtration Rate

### Correlation of copeptin with UACR and eGFR

**Fig. 1:**
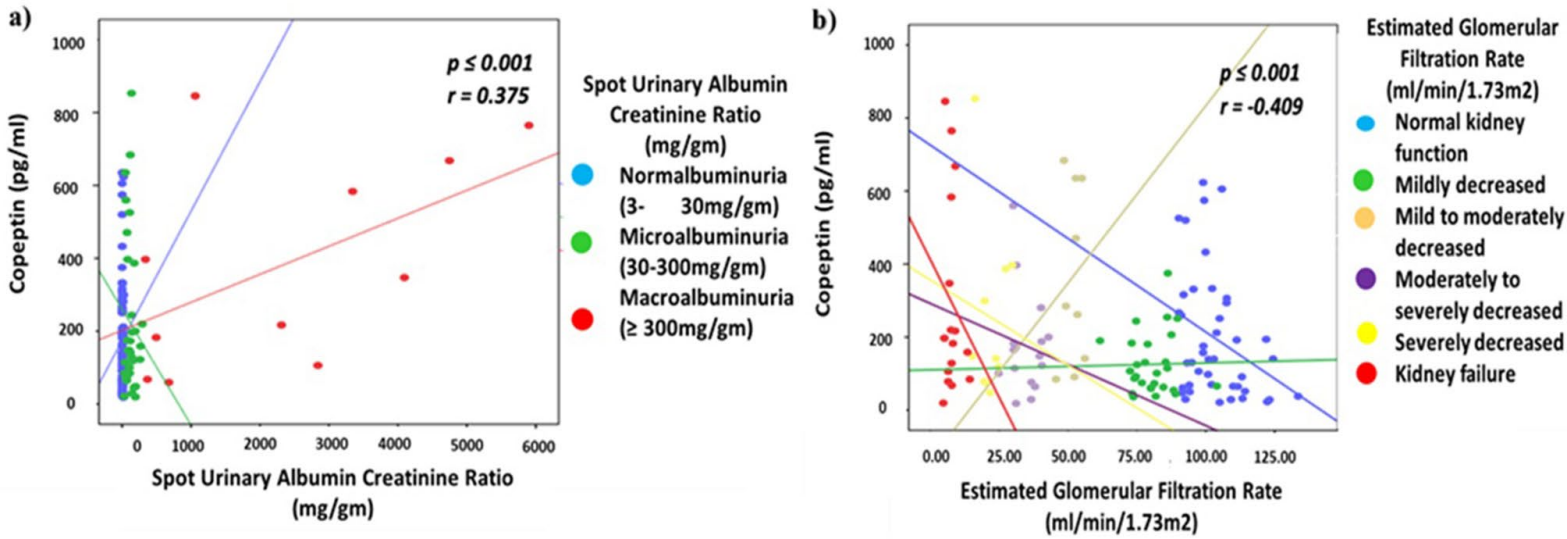
Scatter plots showing Pearson’s correlation coefficient of copeptin with (**a**) UACR for progressive albuminuria and (**b**) GFR for progressive decline in renal function. Significant correlation of serum Copeptin is associated positively with UACR and negatively with e GFR with their progressive stages. Where, UACR = Spot urinary albumin to creatinine ratio and e GFR = Estimated Glomerular Filtration Rate

Significantly positive correlation of copeptin with UACR (*r* = 0.375, p = ≤ 0.001) at 95% confidence interval was found with progressive stages of albuminuria. Similarly, significantly negative correlation of Copeptin was found with eGFR (*r* = -0.409, p = ≤ 0.001) with progressive decline in renal function at 95% confidence interval. The p-value along with Pearson’s correlation coefficient values of copeptin with UACR and eGFR among different stages of albuminuria and kidney failure are given in Fig. 1a and b respectively.

### Multivariate regression model analysis of copeptin with varying outcome variables

The data shows that as patients move from Stage 1 of CKD (Copeptin: 195.8 pg/ml) to Stage 5 (Copeptin: 254.3 pg/ml), the regression model accurately predicts the corresponding surge in serum BUN (up to 137.9 mg/dl) and serum creatinine (up to 6.4 mg/dl). The multivariate linear regression analysis demonstrates that serum copeptin is an independent predictor of renal biomarkers (β = -0.381, *p* ≤ 0.001). The persistence of significant standardized beta values after adjusting for confounders like age, medication and diabetes duration suggests that Copeptin can serve as an early clinical indicator of nephropathy progression toward ESRD as given in Table 4.

**Table 4:** Multivariable regression analysis of serum copeptin as a predictor for renal dysfunction secondarily to type 2 diabetes mellitus

S. no.	Outcome Variable	Unstandardized coefficient (B)	Standard error of β	Standardized β	*p*-value	95% Confidence Interval	
						Upper Bound	Lower Bound
1.	Age (in years)	-0.112	0.054	-0.105	0.039	-1.57	6.16
2.	Duration of DM	-0.215	0.101	-0.198	0.036	-6.330	6.482
3.	Anti-hypertensive medications	0.045	0.032	0.038	0.16	-70.492	98.876
4.	Anti-diabetic medications	0.021	0.015	0.019	0.163	-86.059	53.320
5.	UACR (mg/g)	0.341	0.048	0.380	≤ 0.001	0.246	0.436
6.	Serum BUN (mg/dl)	0.312	0.055	0.345	≤ 0.001	0.204	0.420
7.	Serum Creatinine (mg/dl)	0.325	0.041	0.362	≤ 0.001	0.244	0.406

Where, BUN = Blood Urea Nitrogen, DM = Diabetes Mellitus and UACR = Urine Albumin to Creatinine Ratio

### Receiver operating characteristics curve (ROC) analysis

Receiver Operating Characteristics Curve (ROC) illustrates the performance of serum copeptin as a predictive biomarker of value diabetic nephropathy as shown in Supplementary Fig. 1. The analysis yielded an Area Under Curve (AUC) of 0.73 (95% CI: 0.7–0.8), which was statistically significant (*p* ≤ 0.001), clearly indicating its ability to distinguish between early and late nephropathy due to Type 2 DM. At the determined optimal cutoff of 215.5 pg/ml, copeptin provided an associated sensitivity of 68.9% and a specificity of 72.6%.

## Discussion

The study investigated the association of plasma copeptin levels with worsening renal outcomes in terms of declining GFR and progressive albuminuria in patients with T2DM. This relation of copeptin remained significantly higher even after adjusting several relevant covariates such as age, gender, duration of disease, antidiabetic medications and antihypertensive treatment after applying regression model and Receiver Operating Characteristics Curve (ROC) analysis.

The study also revealed positive correlation of copeptin both with UAE which is not only widely reported clinically as well as UACR which is hallmark for diagnosing DKD [17, 23]. Additionally, BUN and creatinine are also found positively correlated with copeptin levels. Whereas, copeptin levels rise progressively as e GFR decreases with significantly negative correlation. The findings of our study are in line with the results of study conducted by Velho G et al. and Boertien WE et al. [12, 27].

While copeptin’s discriminative performance was exceeded by the standard clinical indices, its significant AUC suggests its potential as a non-inferior circulating predictor of T2DM associated renal injury severity which is equally comparable to other novel biomarkers, gold-standard biomarkers and in line with the available literature [28], thus supporting its inclusion as a supplementary indicator in comprehensive renal risk assessment.

The results of the study were consistent with the previous findings when the levels of copeptin increased progressively across albuminuria and eGFR stages, paralleling standard renal biomarkers making the copeptin highly reliable biomarker for progressive nephropathy secondary to T2DM or other metabolic diseases. This notable finding endorses the role of copeptin is not just diagnostic, but being part of the AVP axis, which is a modifiable therapeutic pathway in preventing nephropathy towards ESRD as also reported previously by Enhorning S and Meijer E [19, 29]. These findings are also in accord with findings reported by Ronnan R and et al. [30], Boustany RE and et al. [31], and Boertin WE and et al. [12].

A spike in the levels of copeptin is however, observed among patients with mild to moderately decreased kidney functions (stage 3a: GFR = 45–59 ml/min/1.73m^2^). This is contrary to the findings reported by Viella Torres et al. who reported higher levels of serum copeptin stage 5 kidney disease patients [13]. It could be due to smaller sample size in stage 3a (moderate to severely decreased kidney function) or due to non-functional AVP system in stage 4 and 5 patients who become dialysis dependent [23]. Moreover, the Asian population might have higher serum copeptin levels, possibly due to dehydration secondarily to hot weather, however, local literature is scarce to relate the findings.

Our study is also associated with certain limitations, such as we could not use gold standard KRYPTOR, due to budget constraint, which had been used in the majority of international studies [18–20] and [21], thereby limiting the ability to directly compare the results with existing literature. Insufficient number of participants in each group, lack of drug matching effects and hemodialysis status could also be the reason for this variation. Further, these findings also cannot be extrapolated to T1DM as they excluded this diagnosis.

Several studies with variable ethnicity and study parameters are available that have reported significant association of copeptin with T2DM and renal outcomes with few biomarkers of renal function [12, 13, 19, 20, 30] and [31]. But the data concerning copeptin levels in T2DM patients from Pakistan is lacking. Being a lower socioeconomic state with higher prevalence of T2DM, this data is of significant importance from both diagnostic and therapeutic point of view as the current study provides not only baseline levels of copeptin of Pakistani population but also first study showing the trend of changes in copeptin levels across different stages of CKD secondary to T2DM.

Studies done previously showing elevated levels of copeptin in metabolic diseases like PCOS, impaired fasting glucoses levels and ischemic heart diseases. Further, tolvaptan, which is AVP antagonist has given promising outcomes in Autosomal Polycystic Kidney Diseases [23, 32–34], clinical trials and cohorts can be designed to see the effects of vaptans on AVP axis in Diabetic Kidney Disease (DKD) as there are significant lacunae of data regarding tolvaptan and incretin trials, copeptin antagonist in T2DM and other metabolic diseases [23, 35] and [36].

Therefore, it is important to evaluate the role of such drugs in minimizing the burden of rapidly progressing nephropathy Further, Copeptin/AVP system being one of the modifiable risk factors of worsening renal outcomes can be a potential target of further research in this domain. Extending the sample size, regular follow-ups, individual drug matching can also be taken into account in future to design a predictive model to assess how well copeptin predicts worsening of renal function among subjects with T2DM. The role of copeptin as potential help for therapy adjustment would need to be further investigated.

## Conclusion

Serum copeptin correlated strongly with albuminuria and eGFR decline in T2DM, supporting its predictive role as biomarker of DKD approaching ESRD. Incorporating copeptin into risk-stratification models may improve early detection and intervention, particularly in high-burden regions such as South Asia. Future multicenter, longitudinal studies and trials with AVP-targeted therapies are required to validate its prognostic and therapeutic utility.

## Supplementary Information

Below is the link to the electronic supplementary material.

Supplementary Material 1

## Data Availability

Data is provided within the manuscript.

## Abbreviations

ANOVA: One-way Analysis of Variance
AVP: Arginine Vasopressin
AUC: Area Under Curve
BUN: Blood Urea Nitrogen
CKD: Chronic Kidney Disease
DM: Diabetes Mellitus
DKD: Diabetic Kidney Disease
DUHS: Dow University of Health Sciences
ELISA: Enzyme Linked Immunosorbent Assay
ESRD: End Stage Renal Disease
GFR: Glomerular Filtration Rate
HbA1c: Glycosylated Hemoglobin
IRB: Institutional Review Board
KDIGO: Kidney Disease: Improving Global Outcome
NKF KDOQI: National Kidney Foundation Kidney Disease Outcome Quality Initiative
PCOS: Polycystic Ovarian Syndrome
ROC: Receiver Operating Characteristics Curve
S.D: Standard Deviation
SPSS: Statistical Program for Social Sciences
T2DM: Type 2 Diabetes Mellitus
UACR: Urinary Albumin Creatinine Ratio
UAE: Urinary Albumin Excretion
