# Supplementary figures and images for: Predictive role and clinical correlation of copeptin in patients with type 2 diabetes mellitus associated nephropathy approaching end-stage renal disease
Source: BMC Nephrol. 2026 Jan 30;27:145. doi: 10.1186/s12882-026-04777-5 (PMC12933918; doi:10.1186/s12882-026-04777-5)

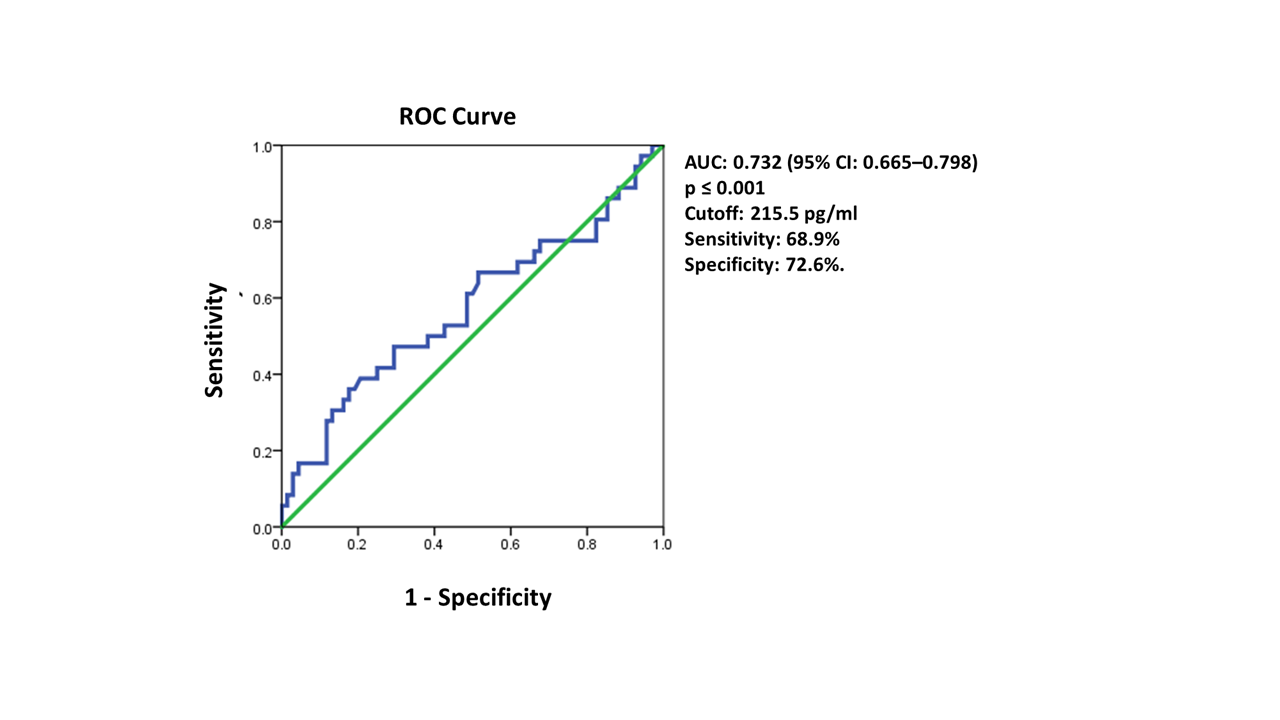

Supplement: Supplementary file 1 — Supplementary Material 1 [file 12882_2026_4777_MOESM1_ESM.png]
